# Neonatal Abstinence Syndrome: Prevention, Management and Outcomes: From Birth to Adulthood

**DOI:** 10.3390/children9081151

**Published:** 2022-07-30

**Authors:** Karel Allegaert, Ju-Lee Oei

**Affiliations:** 1 Child and Youth Institute, KU Leuven, Herestraat 49, 3000 Leuven, Belgium; 2 Department of Development and Regeneration, KU Leuven, 3000 Leuven, Belgium; 3 Department of Pharmaceutical and Pharmacological Sciences, KU Leuven, 3000 Leuven, Belgium; 4 Department of Hospital Pharmacy, Erasmus MC, 3000 GA Rotterdam, The Netherlands; 5 Leuven Child and Youth Institute, KU Leuven, 3000 Leuven, Belgium; 6 School of Women’s and Children’s Health, University of New South Wales, Kensington, NSW 2031, Australia; j.oei@unsw.edu.au

Neonatal abstinence syndrome (NAS), or—when specifically focused on opioids—neonatal opioid withdrawal syndrome (NOWS) is a withdrawal syndrome in neonates after birth causally related to the in utero exposure to drugs of dependence, and the subsequent acute interruption at delivery. NAS is diagnosed by a detailed medication history, further supported by NAS scores to quantify its presence and severity, while additional diagnostics can be considered. This Special Issue on neonatal abstinence syndrome (NAS) explores the consequences of prenatal drug exposure, preferably beyond the immediate neonatal period. This is relevant as NAS has the potential to affect a child even after resolution of withdrawal, precipitating a gamut of biological, genetic and societal changes that may, more often than not, result in detrimental outcomes for not only the child but also their family and the whole society. This Special Issue intended to encourage clinicians, families, and policymakers to consider strategies that will minimize and reduce the impact of NAS on both an individual and a societal level. We hereby can cluster the published papers in three interrelated topics (Figure 1).

The papers either relate to diagnosis (assessment, methadone initiation based on PK dosing regimens), to clinical management, with consecutive papers focused on nurses and midwives (qualitative research on nursing themes), parents (how to promote parental attachment) and foster carers (how foster carers perceive the use of expressed breast milk (EBM)), or to economics and quality of life outcomes (cost effectiveness of (non)-pharmacological interventions, preschool language performance and its modulators). 

Symptoms of NAS manifest from the central and autonomic nervous systems as well as the gastrointestinal system and vary in severity and duration. The clinical management of infants—including assessment—experiencing NAS is dependent on symptoms and may include both pharmacological and non-pharmacological measures, with nurses or midwives involved in its assessment. The Finnegan score is the most widely used tool to assess NAS, while others consider, e.g., the Neonatal Network Neurobehavioral Scale (NNNS). In a survey on the use of both scales by 41 nurses on 78 newborns exposed to methadone, the correlation between both scores remained rather weak, and nurses reported that the Finnegan score was perceived to be somewhat to very subjective. An NNNS-based assessment of NAS could quantify specific strengths and weaknesses at the level of the individual infant that could identify functional neurobehavioral domains to target for intervention. Sharing this kind of information with caregivers could also strengthen the developing infant–caregiver relationship and provide the bases for additional non-pharmacological care [1]. In a second paper, Samiee-Zafarghandy et al. illustrated the add-on value of simulations of methadone exposure (based on existing population pharmacokinetic data) and methadone umbilical cord blood concentrations for early identification of those at risk of severe NAS and for designing a methadone dosing regimen that can provide safe and effective drug exposure [2]. 

Related to clinical management, there are three papers with focus on different care providers involved in this care process, i.e., nurses and midwives, parents and foster carers respectively [3,4,5]. Shannon et al. collected nurses’ and midwives’ experiences of delivering care for infants with NAS, using semi-structured interviews. Five themes emerged: complex care needs; prioritizing physiological care; experiencing compassion fatigue; lacking continuity of care; and stigma. The authors hereby concluded that these five nursing themes demonstrated the complex nature of care provision for NAS infants. Competing priorities and the stigmatizing nature of NAS threaten optimal care being delivered to these vulnerable infants and their parents [3]. Attachment for hospitalized NAS neonates with their mothers has its challenges, as mothers with a history of substance abuse are likely less sensitive to their infants’ cues. Facilitation of the attachment relationship was mainly promoted when the mother was present. However, parents were often reported to be absent from the nursery. Difficulties in promoting an attachment relationship were also identified when an infant had child protection involvement [4]. Foster carers’ views and concerns relate to this child protection involvement, so that Blythe et al. reported on an online survey on how foster carers perceive the use of EBM in this specific setting. Interestingly, foster carers were generally open to the idea of maternal breastfeeding and infants in their care receiving EBM from their mothers. However, the majority of respondents expressed concern regarding the safety of EBM for infant consumption due to the possibility of harmful substances in the milk. Concerns regarding the safety of handling EBM were also prevalent. These concerns caused foster carers to discard EBM. Findings suggest foster carers may lack knowledge related to maternal substance use and breastmilk. At least, these results provide the information to develop solutions for EBM [5].

The last part of the Special Issue has its focus on issues related to economics and quality of life, and the economic evidence on the (non)pharmacological management of infants with NOWS. Based on a systematic assessment of the literature, the authors highlighted the paucity of high-quality studies assessing the cost-effectiveness of intervention strategies for NOWS. There is also a lack of evidence on long-term outcomes associated with NOWS and its treatment. The authors therefore concluded that inclusion of economic analyses in future studies is needed to provide evidence to inform policymakers on resource allocation decisions for this patient population [6]. Finally, Kim et al. focused on preschool language development in NOWS children. Using the Clinical Evaluation of Language Fundamentals (CELF-P) scale at 4.5 years in 89 opioid exposed and 103 non-exposed controls, opioid exposure was an independent predictor of the CELF-P score, with the quality of parenting and home environment at 18 months, and early childhood education participation being positive mediators [7].

In conclusion, this Special Issue has highlighted recent progress and knowledge gaps that should be addressed in the near future to further improve NAS management, including the need for long-term outcome data and inclusion of caregivers and allied health professionals in providing research and practice guidance for this complex and growing group of vulnerable infants. 

## Figures and Tables

**Figure 1:**
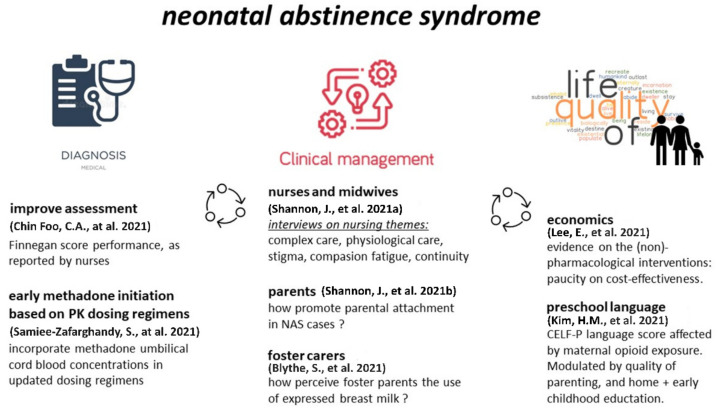
Overview of the themes and specific papers discussed in this Special Issue [1,2,3,4,5,6,7].

## Data Availability

Not applicable.

## References

[1] Chin Foo C.A., Dansereau L.M., Hawes K., Oliveira E.L., Lester B.M. (2021). Improving the Assessment of Neonatal Abstinence Syndrome (NAS). Children.

[2] Samiee-Zafarghandy S., van Donge T., Allegaert K., van den Anker J. (2021). Pharmacometric Evaluation of Umbilical Cord Blood Concentration-Based Early Initiation of Treatment in Methadone-Exposed Preterm Neonates. Children.

[3] Shannon J., Blythe S., Peters K. (2021). The Complexities Associated with Caring for Hospitalised Infants with Neonatal Abstinence Syndrome: The Perspectives of Nurses and Midwives. Children.

[4] Shannon J., Peters K., Blythe S. (2021). The Challenges to Promoting Attachment for Hospitalised Infants with NAS. Children.

[5] Blythe S., Peters K., Elcombe E., Burns E., Gribble K. (2021). Australian Foster Carers’ Views and Concerns Regarding Maternal Drug Use and the Safety of Breastmilk. Children.

[6] Lee E., Schofield D., Azim S.I., Oei J.L. (2021). Economic Evaluation of Interventions for Treatment of Neonatal Opioid Withdrawal Syndrome: A Review. Children.

[7] Kim H.M., Bone R.M., McNeill B., Lee S.J., Gillon G., Woodward L.J. (2021). Preschool Language Development of Children Born to Women with an Opioid Use Disorder. Children.

